# Supplementary material for: Systematic Construction and Validation of a Prognostic Model for Hepatocellular Carcinoma Based on Immune-Related Genes
Source: Front Cell Dev Biol. 2021 Oct 4;9:700553. doi: 10.3389/fcell.2021.700553 (PMC8520962; doi:10.3389/fcell.2021.700553)
Supplement: Supplementary file 1 [file Data_Sheet_1.docx]

**Supplementary Figure 1. The flow chart of the current study.**


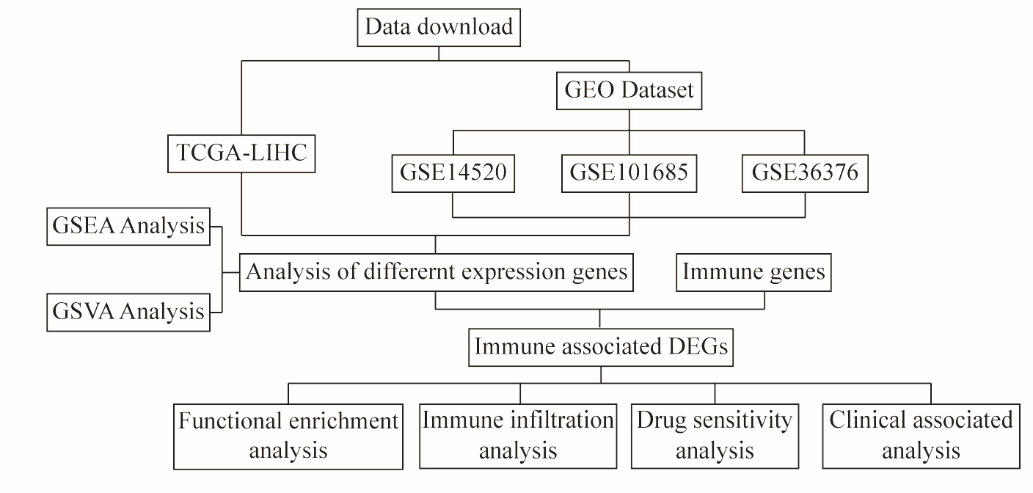


**Supplementary Figure 2. The principal component analysis (PCA) of four datasets.** Based on the gene expression data of HCC patients, the overall differences between tumor tissues and adjacent normal tissues in four different datasets were analyzed using PCA. (A) the TCGA-HCC dataset; (B) the GSE14520 dataset; (C) the GSE101685 dataset; (D) the GSE36376 dataset.


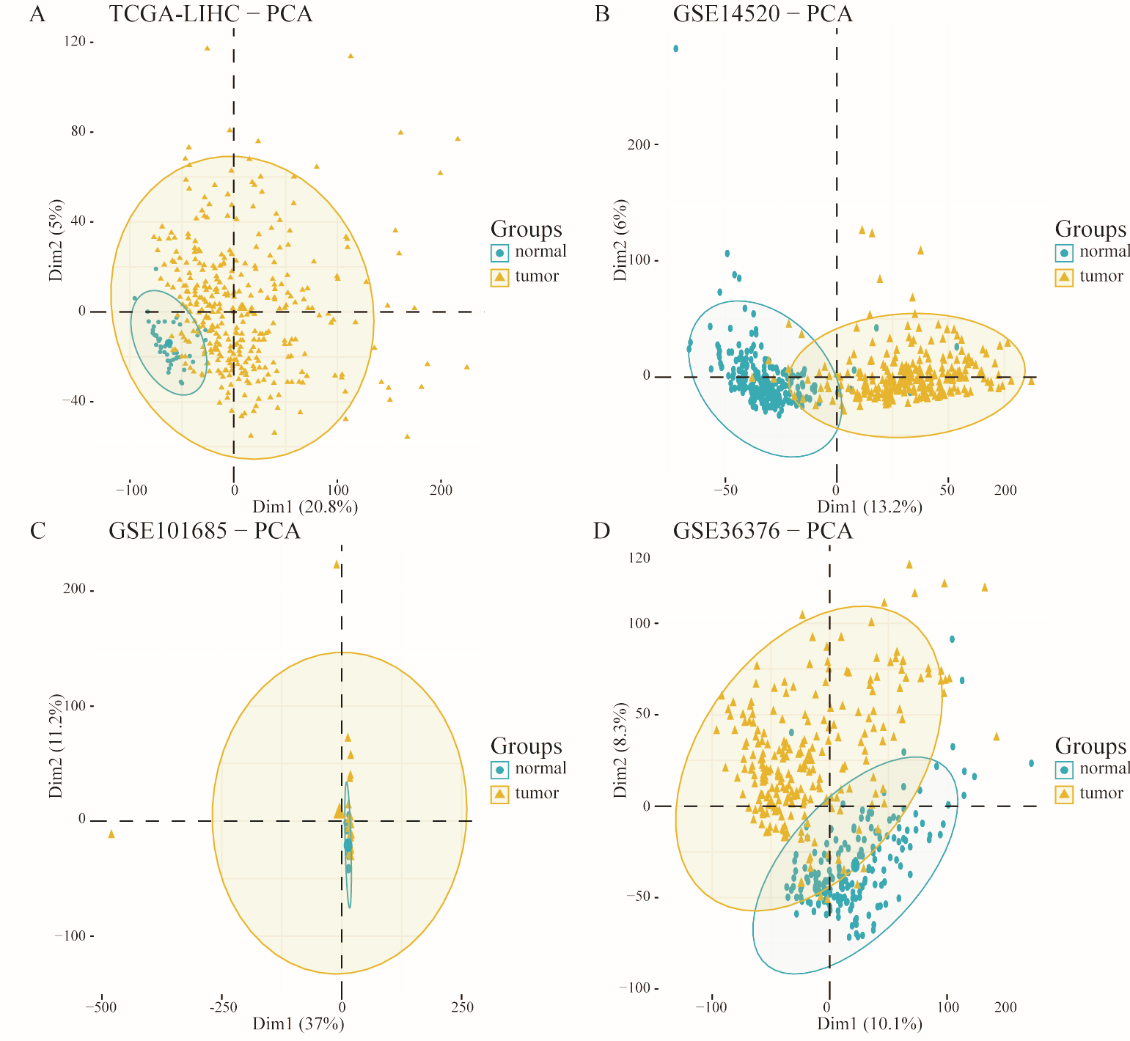


**Supplementary Figure 3.** **Venn diagram analysis of the common differentially expressed IRGs.** (A) Among the 4 datasets, there were 206 common differentially expressed genes. (B) From the 206 common differentially expressed genes and the immune-related genes from ImmPort database, 20 common differentially expressed immune-related genes were identified.

**
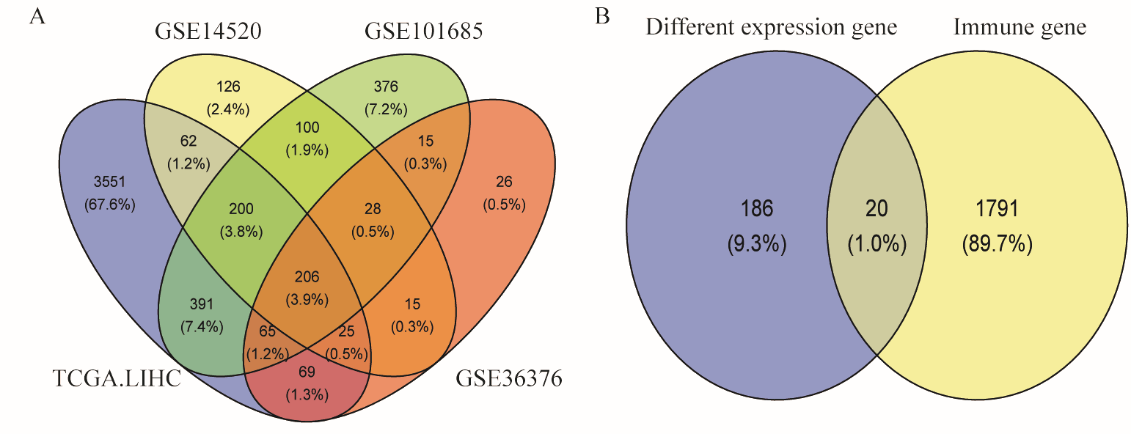
**

**Supplementary Figure 4. The gene set enrichment analysis (GSEA) of TCGA-HCC dataset.** (A) Volcano plot showed GSEA based on the differential expression of TCGA-HCC dataset; (B) Results of GSEA showed that HCC tissues are closely associated with ribosome, complement and coagulation cascades, PPAR signaling pathway and glycolysis, gluconeogenesis.


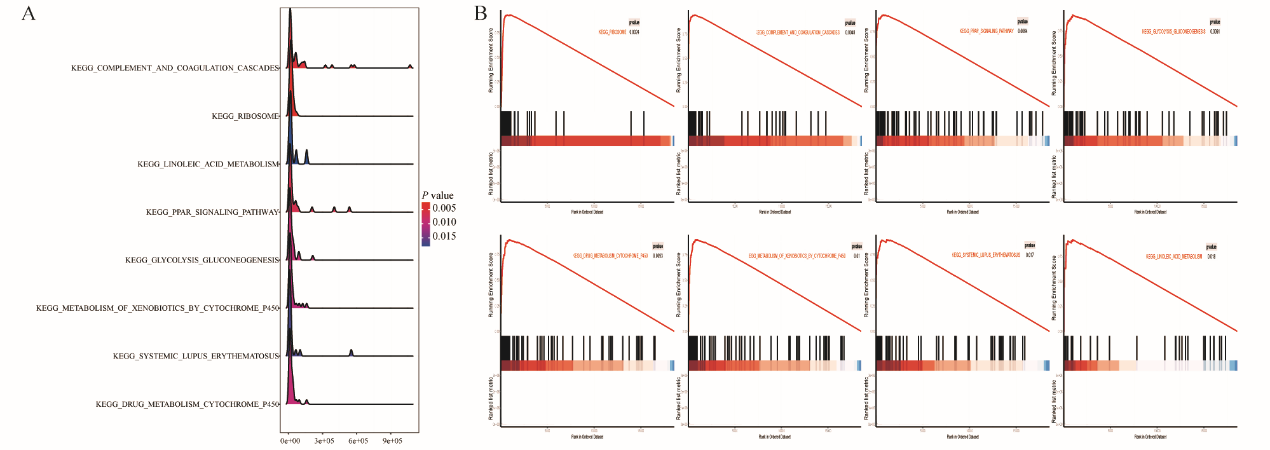


**Supplementary Figure 5. The demonstration of Gene set variation analysis (GSVA) of tumor and adjacent normal tissues in HCC patients.** GSVA was performed on tumor and adjacent normal tissues based on gene expression data from HCC patients, and heat maps were used to demonstrate relevant pathways with significant differential enrichment. (A) the TCGA-HCC dataset; (B) the GSE14520 dataset; (C) the GSE101685 dataset; (D) the GSE36376 dataset.


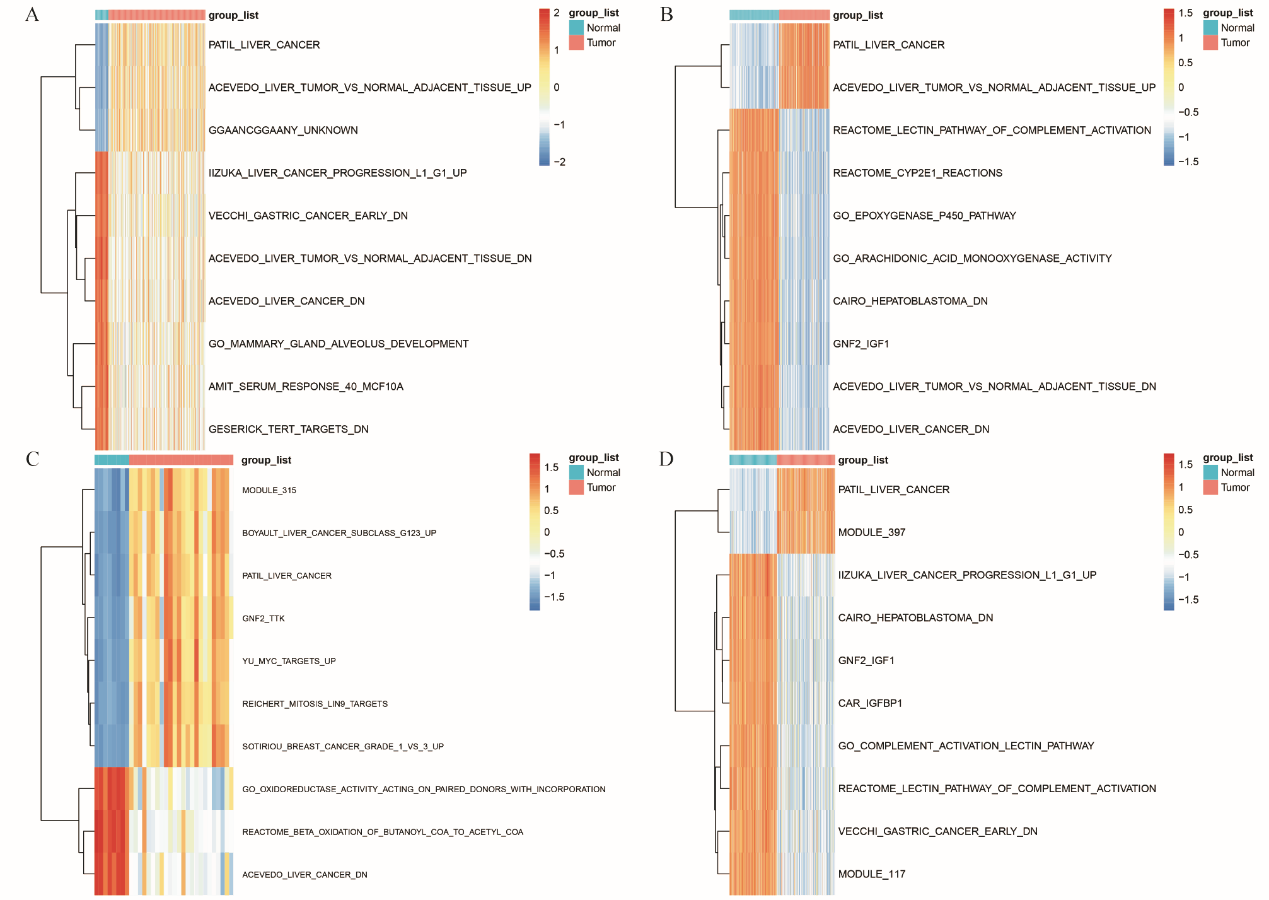


**Supplementary Figure 6. Construction of the PPI network.** (A)A PPI network was constructed from the 20 differentially expressed immune-related genes using STRING database, where color shades are proportional to logFC; (B) The 20 differentially expressed immune-related genes were incorporated, and the top 6 genes were selected as characteristic genes based on the MCC algorithm for further study.
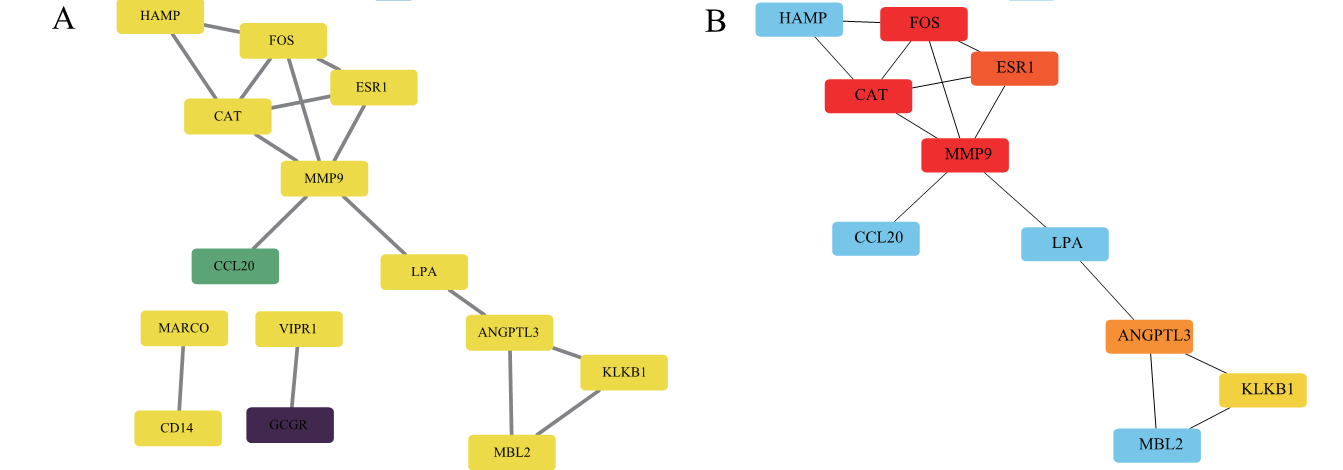


**Supplementary Figure 7. The gene set enrichment analysis of 6 characteristic IRGs**

**
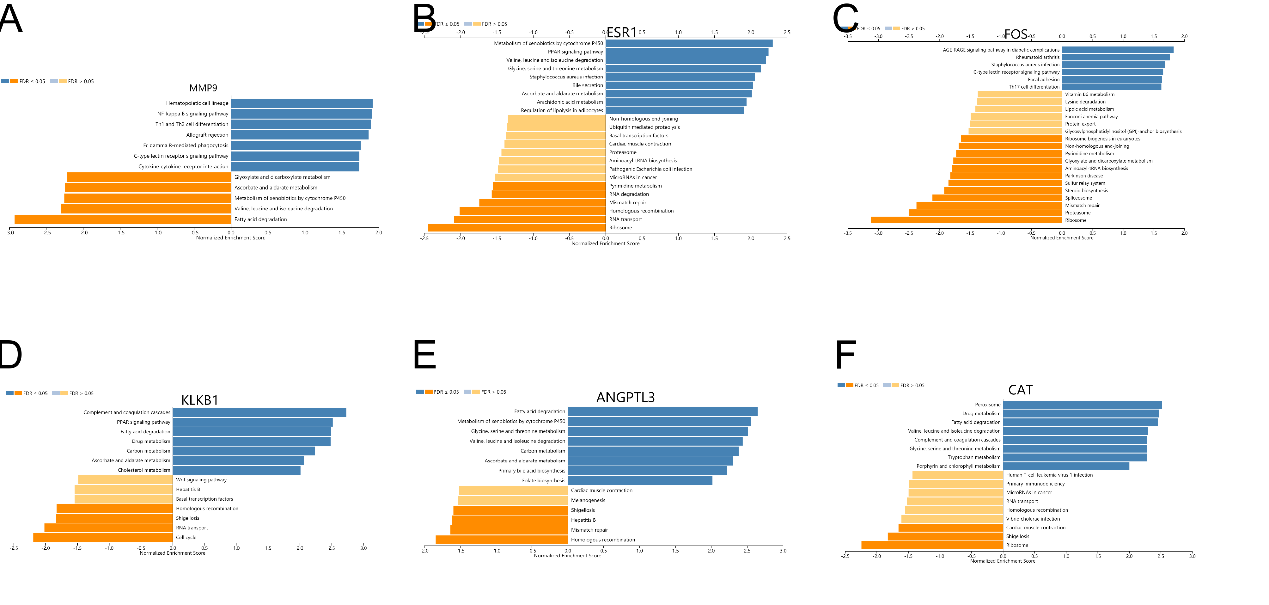
**

**Supplementary Figure 8. The miRNA-mRNA network construction of 20 differential immune-related genes.** (A) miRNA-mRNA regulatory network was constructed by miRTarBase database. (B) The transcriptional regulatory network of 20 differentially expressed immune-related genes, of which green is the differential immune genes.


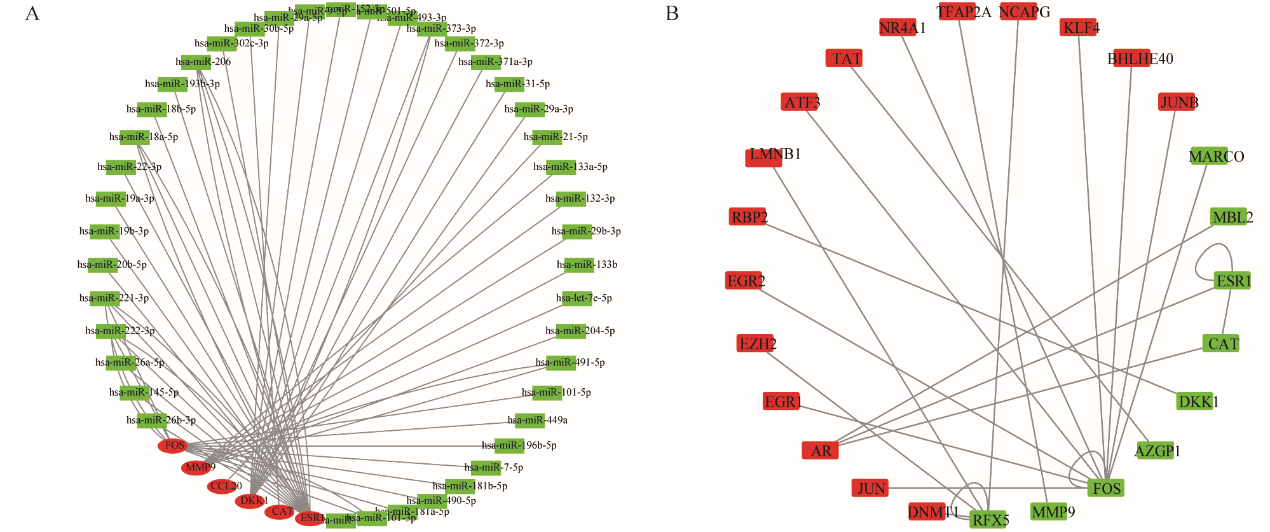


**Supplementary Figure 9. The expression of 6 characteristic IRGs in different cancers.**

**
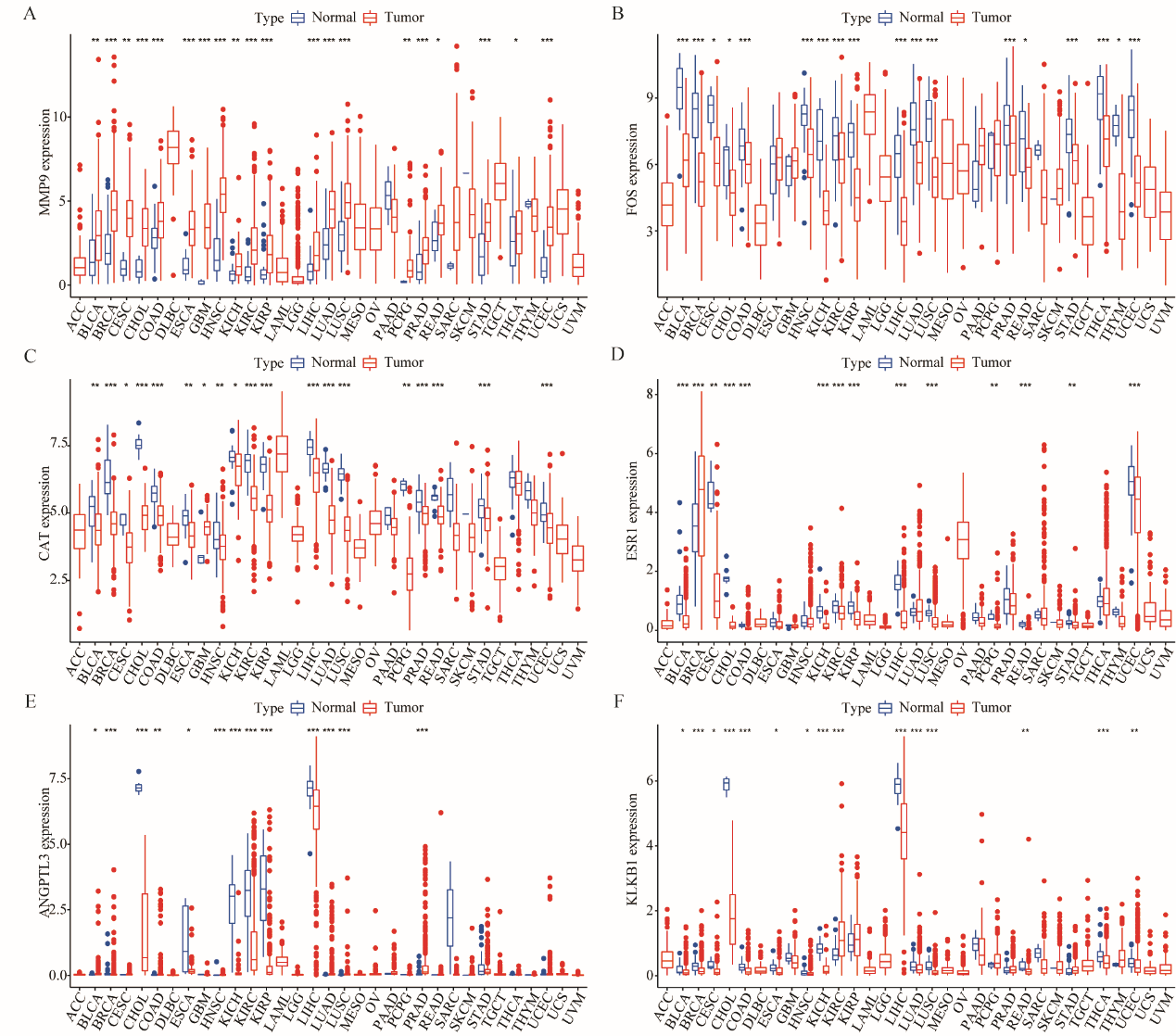
**

**Supplementary Figure 10. The expression of 6 characteristic IRGs in different cell lines.**

**
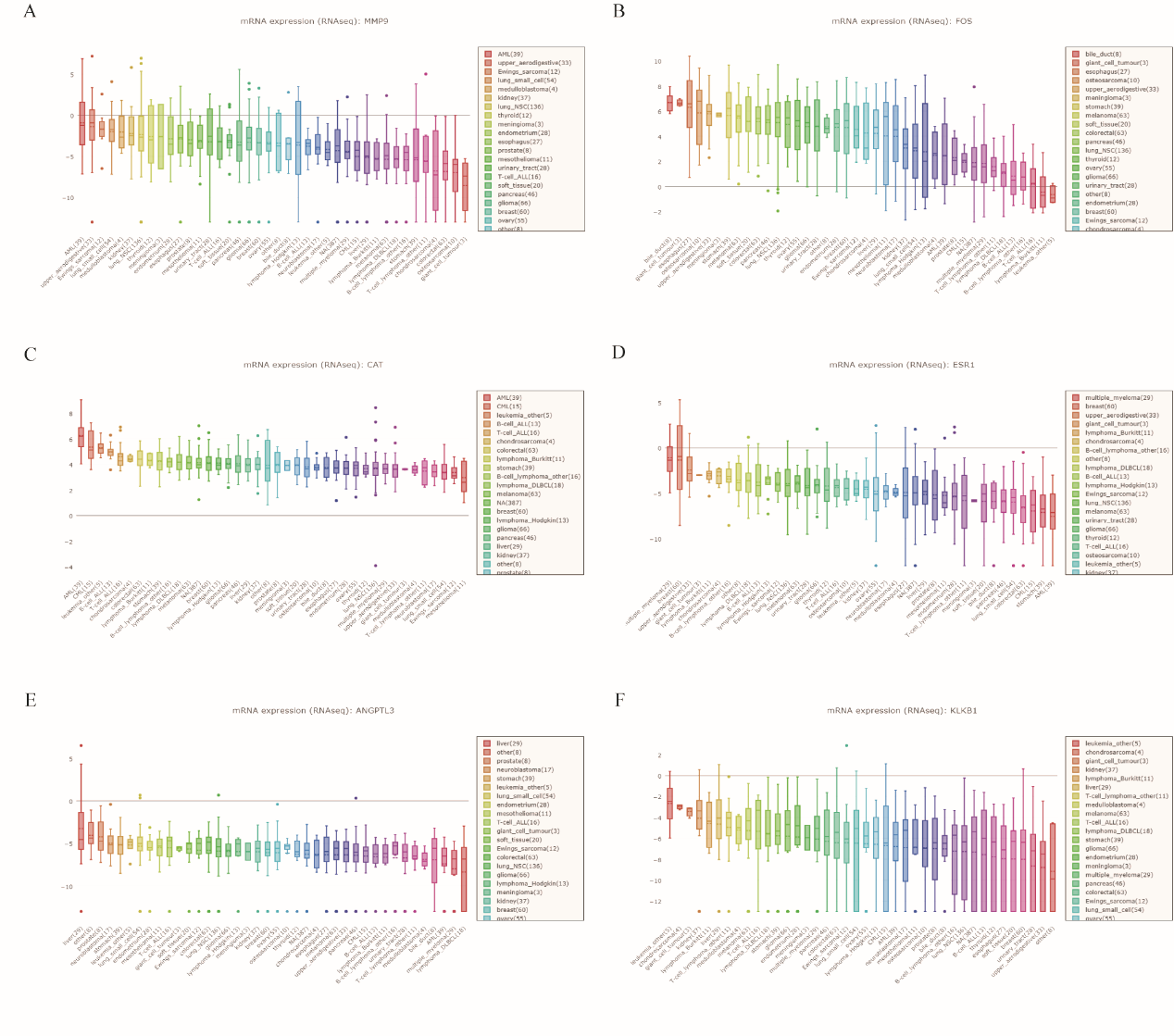
**

**Supplementary Figure 11. The ROC analysis of 6 characteristic IRGs.** The results showed that all 6 characteristic IRGs were able to classify HCC tissues and adjacent normal tissues.


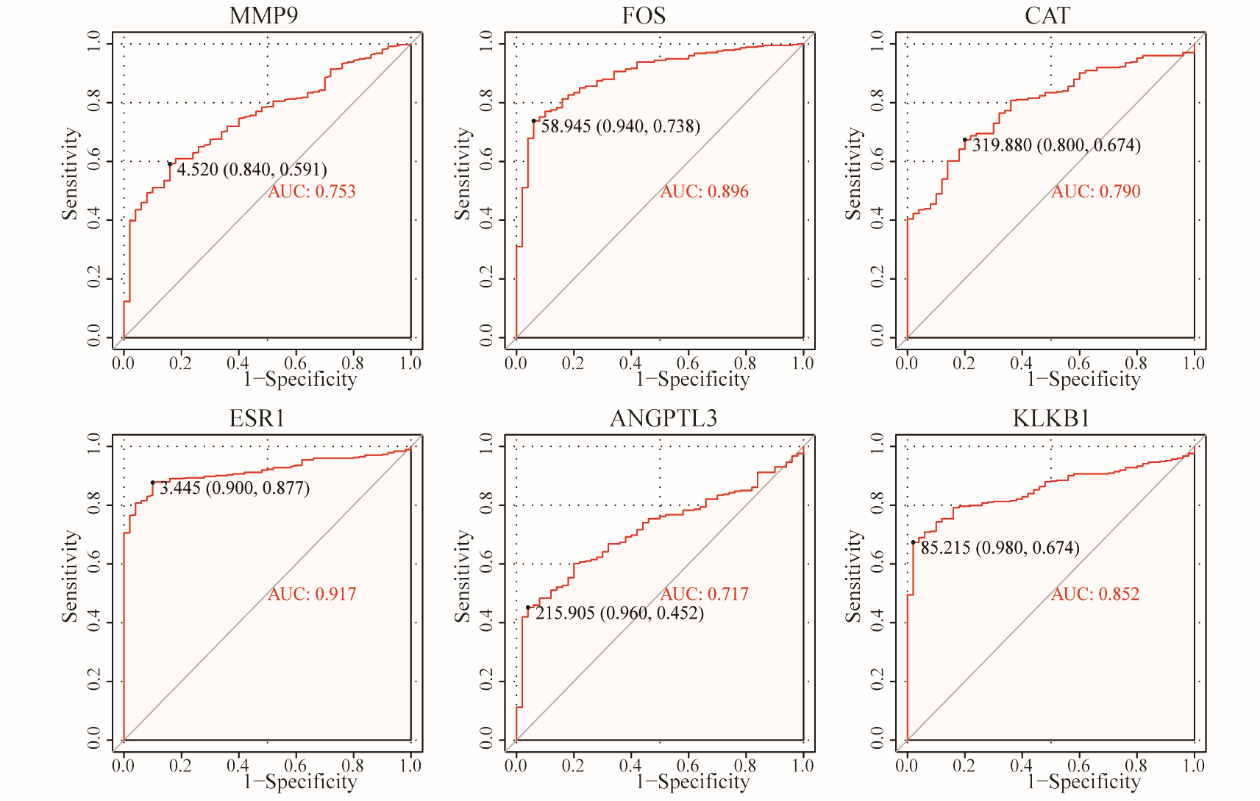


**Supplementary Figure 12. The analysis of sensitivity of 6 characteristic IRGs to anticancer drugs based on CellMiner database.**

**
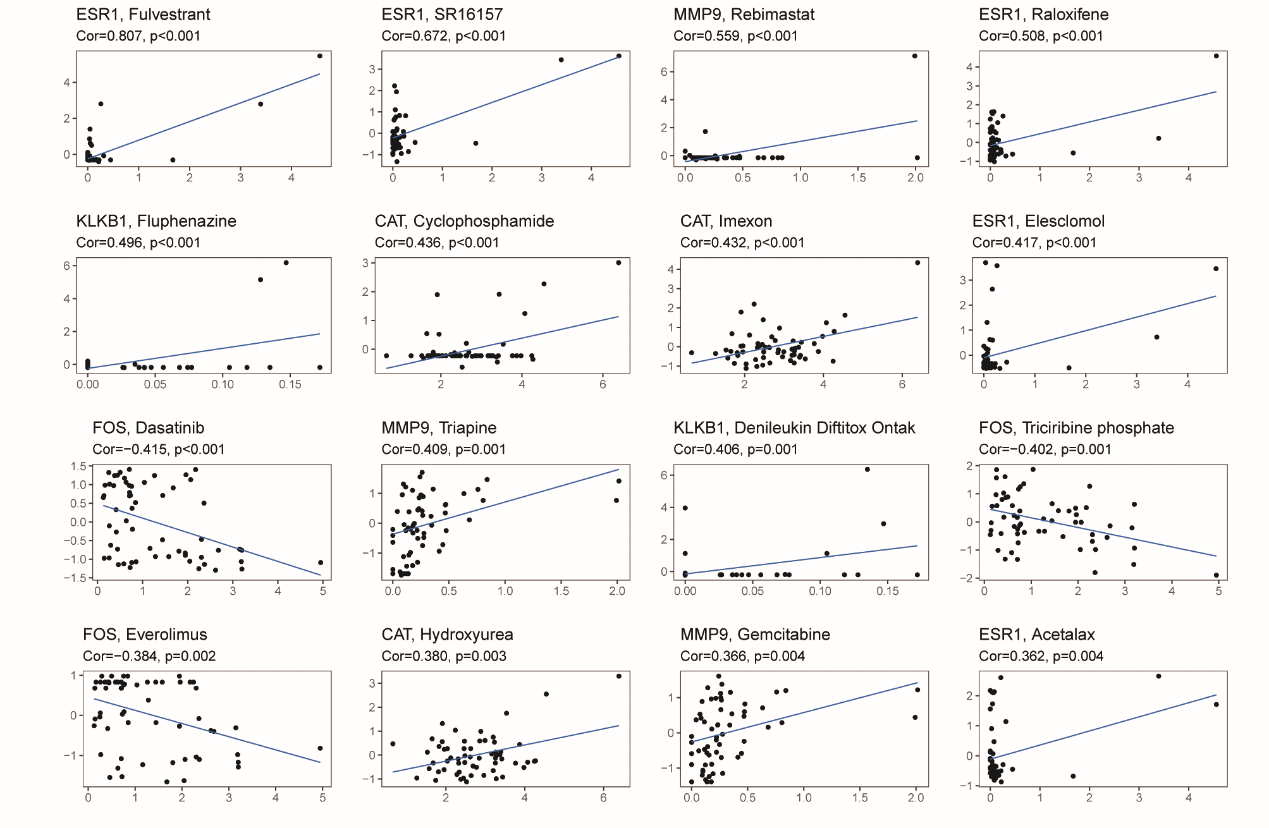
**

**Supplementary Figure 13. The correlation of 6 characteristic IRGs with PD-1, PD-L1, CTLA-4.**

**
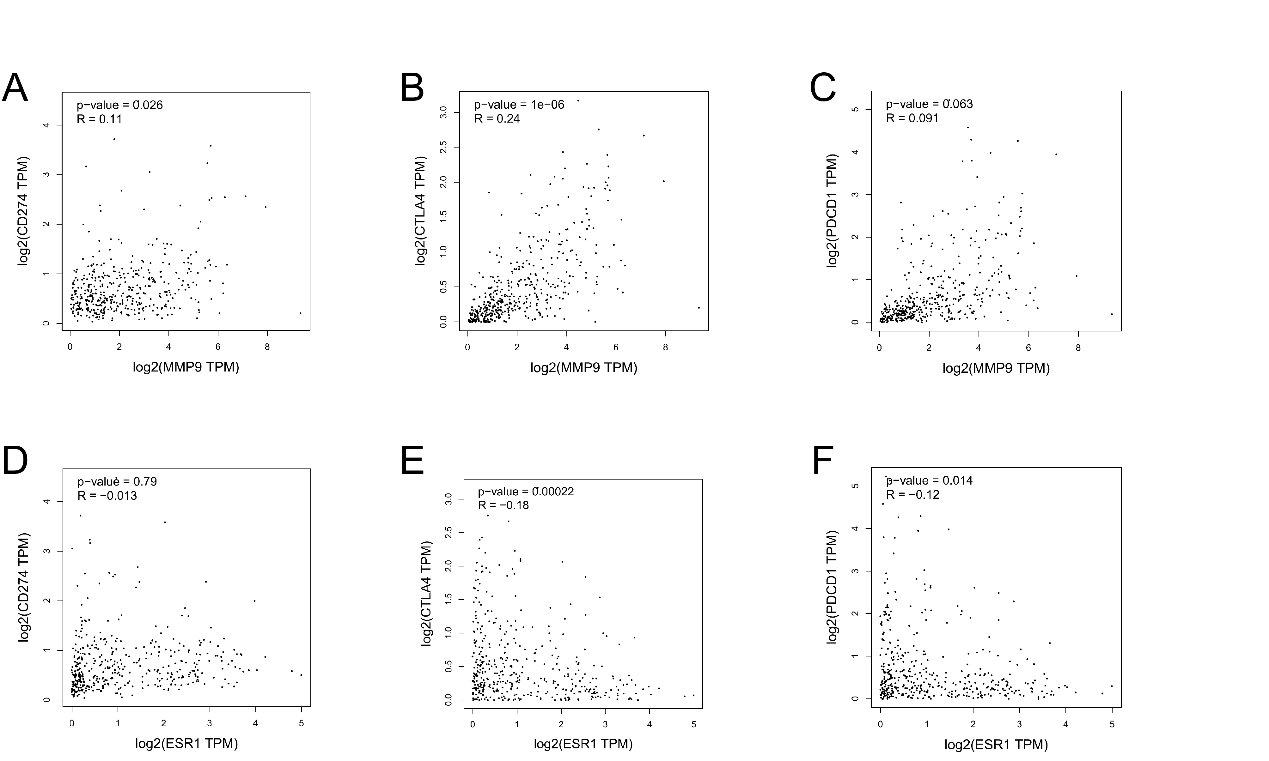
**

**Supplementary Figure 14. External Validation of the prognostic models using the data from ICGC.**

**
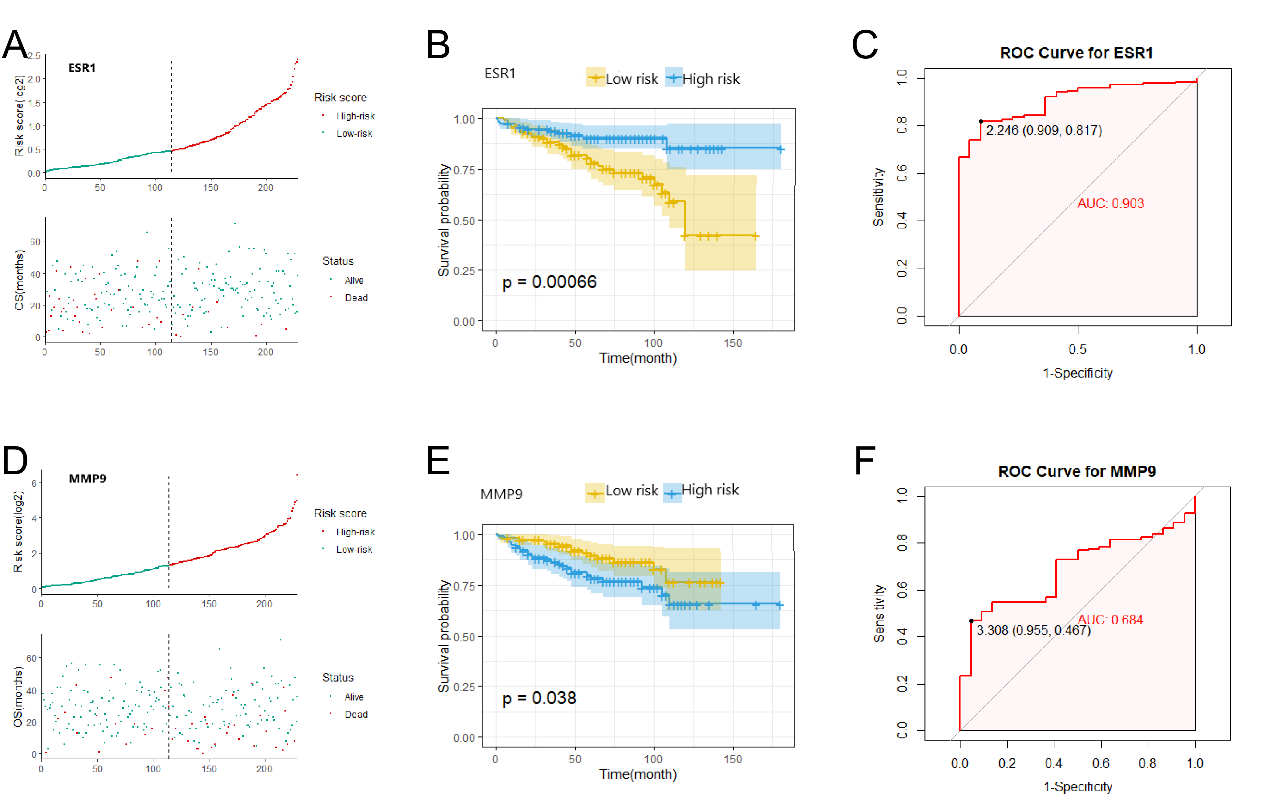
**

**Supplementary Table 1. The results of GO analysis of 20 differential expressed immune-related genes.**

| ONTOLOGY | ID | Description | Count | P value | Gene |
| --- | --- | --- | --- | --- | --- |
| BP | GO:0010038 | response to metal ion | 5 | 3.25E-05 | FOS/HAMP/CD14/CAT/MMP9 |
| BP | GO:0046686 | response to cadmium ion | 3 | 4.01E-05 | FOS/CAT/MMP9 |
| BP | GO:0097305 | response to alcohol | 4 | 9.79E-05 | FOS/HAMP/CD14/CAT |
| CC | GO:0070195 | growth hormone receptor complex | 1 | 0.002028 | GHR |
| CC | GO:0005581 | collagen trimer | 2 | 0.003473 | MARCO/MBL2 |
| CC | GO:0035976 | transcription factor AP-1 complex | 1 | 0.004052 | FOS |
| MF | GO:0042277 | peptide binding | 5 | 1.57E-05 | VIPR1/MARCO/GCGR/GHR/CD14 |
| MF | GO:0017046 | peptide hormone binding | 3 | 2.20E-05 | VIPR1/GCGR/GHR |
| MF | GO:0033218 | amide binding | 5 | 3.87E-05 | VIPR1/MARCO/GCGR/GHR/CD14 |

**Supplementary Table 2. The results of KEGG analysis of differential expressed immune-related genes.**

| ID | Description | Count | P value | Gene |
| --- | --- | --- | --- | --- |
| hsa04657 | IL-17 signaling pathway | 3 | 0.000919 | FOS/CCL20/MMP9 |
| hsa01522 | Endocrine resistance | 3 | 0.001037 | ESR1/FOS/MMP9 |
| hsa04668 | TNF signaling pathway | 3 | 0.001526 | FOS/CCL20/MMP9 |
| hsa04915 | Estrogen signaling pathway | 3 | 0.002775 | ESR1/FOS/MMP9 |
| hsa04145 | Phagosome | 3 | 0.003648 | MARCO/CD14/MBL2 |
| hsa04979 | Cholesterol metabolism | 2 | 0.004802 | LPA/ANGPTL3 |
| hsa04917 | Prolactin signaling pathway | 2 | 0.009236 | ESR1/FOS |
| hsa05133 | Pertussis | 2 | 0.01082 | FOS/CD14 |
| hsa04610 | Complement and coagulation cascades | 2 | 0.013404 | KLKB1/MBL2 |
